# Knockdown of DEAD-box RNA helicase 52 (DDX52) suppresses the proliferation of melanoma cells in vitro and of nude mouse xenografts by targeting c-Myc

**DOI:** 10.1080/21655979.2021.1950283

**Published:** 2021-07-07

**Authors:** Qiang Wang, Leqi Qian, Mengyuan Tao, Jiaqi Liu, Fa-zhi Qi

**Affiliations:** Department of Plastic Surgery, Zhongshan Hospital, Fudan University, Shanghai, China

**Keywords:** Melanoma, DDX52, c-Myc, proliferation

## Abstract

The ATP-dependent protein DEAD-box RNA helicase 52 (DDX52) is an important regulator in RNA biology and has been implicated in the development of prostate and lung cancer. However, its biological functions and clinical importance in malignant melanoma (MM) are still unclear. Understanding the potential mechanism underlying the regulation of MM progression by DDX52 might lead to novel therapeutic strategies. The aim of the present study was to investigate the role of DDX52 in the regulation of MM progression and its clinical relevance. DDX52 expression in normal and MM tissues was evaluated by GEO analysis and immunohistochemistry. The effects of DDX52 on cell growth were evaluated in MM cells with downregulated DDX52 expression. In this study, we found that DDX52 was markedly overexpressed in MM tissues compared with nontumor tissues and was associated with shorter overall survival in patients; therefore, DDX52 might be a prognostic marker in MM. Downregulation of DDX52 expression in the MM cell lines A2058 and MV3 markedly inhibited cell proliferation and colony formation. Additionally, knockdown of DDX52 in MM cells caused significant regression of established tumors in nude mice and delayed the onset time. Moreover, downregulation of DDX52 markedly suppressed c-Myc mRNA and protein expression, and an RNA immunoprecipitation assay confirmed the association between DDX52 and c-Myc. Restoration of c-Myc expression partly rescued the effects of DDX52 deficiency in MM cells. In conclusion, our study found that DDX52 mediated oncogenesis by promoting the transcriptional activity of c-Myc and could be a therapeutic target in MM.

## Graphical Abstract

**Figure:**
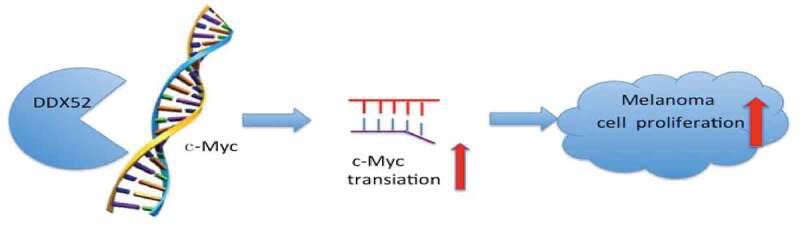

## Introduction

Malignant melanoma (MM) is an aggressive cancer, and its incidence is increasing [1]. More than 100,000 global cases of MM are estimated to have been diagnosed in 2020 [2]. Although immune therapy and BRAF inhibitors have improved survival, the majority of patients with MM eventually relapse and develop drug resistance [3]. Therefore, it is important to identify novel biomarkers and clarify the molecular mechanism underlying the progression of MM.

DEAD-box RNA helicase 52 (DDX52), also known as ROK1, is a member of the DEAD-box RNA helicase family [4]. Members of this family are involved in various RNA-based processes and ATP binding and are associated with a variety of human diseases, including disordered spermatogenesis, viral infections and cancer [5–7]. Previous studies showed that RNA helicases could act as oncogenes and had upregulated expression during cancer progression. Several DEAD-box RNA helicases regulate the progression of MM. For example, DDX39 is overexpressed in MM tissues, and its downregulation inhibits the proliferation and invasion of melanoma cells [8]; In WM1158 melanoma cells, disruption of DDX11 expression induces apoptosis [9]. Additionally, the levels of chromatin-bound DDX21 were reduced in nucleotide-depleted A375 melanoma cells, restricting the differentiation of melanocyte stem cells [10,11]. Therefore, blockade of DEAD-box RNA helicases shows therapeutic potential for cancer. Previous studies have shown that DDX52 expression is downregulated by activin-induced inhibition of the proliferation of prostate cancer cells and is associated with lung cancer [12,13]; however, the clinical and biological functions of DDX52 in MM are unknown.

In the present study, we hypothesized that DDX52 is involved in the progression of MM by activating c-Myc. To prove this hypothesis, we detected the expression of DDX52 in human melanoma tissues and evaluated its effects on melanoma cell growth in vivo and in vitro. Additionally, the relationship between DDX52 and c-Myc in MM was investigated.

## Methods and materials

### Tissue specimens and melanoma cell lines

The human melanoma cell lines A375, A2058, and MV3 were obtained from American Type Culture Collection (ATCC, USA). Cells were cultured in DMEM medium (HyClone, USA) supplemented with 10% FBS (HyClone, USA). Cells were maintained at 37°C in humidified conditions with 5% CO2.

The human melanoma tissue microarray (ME1004g) used in this study was purchased from Xi’an AlenaBio Technology Company (Shanxi, China). It contains 62 samples of primary melanoma, 20 samples of metastatic melanoma tissues and 18 samples of tissues nevi. Detailed information on this human melanoma tissue microarray is available at www.alenabio.com.

### Immunohistochemistry

A human melanoma tissue microarray was stained as previously described [14]. Briefly, the tissue microarray was deparaffinized in xylene and rehydrated in a standard alcohol gradient. Then the antigen retrieval was performed in in 1 mM EDTA buffer (pH 8.0) for 30 min, followed by incubation of the microarray in 3% H2O2 to block endogenous peroxidase activity. After the samples were blocked in goat serum, DDX52 antibody (1:200) was incubated at 4°C overnight before HRP-conjugated secondary antibody was incubated for 60 min. Then, DAB was used, followed by hematoxylin staining. The IHC scores were calculated as previously described [15].

### Western blot

Western blotting was performed as previously described [15]. Cell lysates were homogenized in RIPA buffer with inhibitors, and the protein concentrations were then quantified by BAC. Then, the lysates were subjected to SDS-PAGE followed by transfer onto a PVDF membrane. The primary antibodies used were as follows: anti-DDX52 (NBP1-71,811, NOVUS), anti-β-actin (60,008–1, Proteintech), anti-c-myc (ab32072, Abcam), and anti-Flag (66,008–3, Proteintech). The secondary antibodies used were as follows: anti-rabbit (7404, CST) and anti-mouse (7076, CST). The immunoreactive bands were captured using a Tanon 5200 system (Tanon, Shanghai, China) with ECL Western blotting Substrate (WBULS0500, Millipore).

## RT-PCR

Total RNA was isolated using TRIzol reagent (Invitrogen, Carlsbad, CA, USA) according to the manufacturer’s instructions. cDNA was synthesized using a PrimeScript RT Master Kit (TaKaRa, Dalian, China). SYBR Green PCR Master Mix was used for qRT-PCR on an ABI7500 System (Applied Biosystems, Foster City, CA, USA). The primer sequences used for qRT-PCR were as follows: GAPDH forward 5′-CTGAGAACGGGAAGCTTGT-3′ and reverse 5′-GGGTGCTAAGCAGTTGGT-3′; DDX52 forward 5′-CTTCTGGCTTCTGCTCCA ACTGG-3′ and reverse 5′-TGTGAATCTGGCTGGCAAGTTCTC −3′; and c-Myc forward 5′-CGACGAGACCTTCATCAAAAAC-3′ and reverse 5′-CTTCTCTGAGACGAGCTTGG-3′. All mRNAs were normalized to the level of GAPDH gene expression.

### Knockdown and overexpression of DDX52

The sequence of shRNA targeting DDX52 is GCAGGTTTCCAAATGCCTACG. The plasmids were transfected into 293 T cells using Lipofectamine 2000 (Invitrogen, Carlsbad, USA) according to the manufacturer’s instructions. A2058 and MV3 cells were transfected with shddx52 or plvx-ddx52-flag and then cultured with puromycin (3 mg/l) for 5 days.

### Cell viability assay

Cell proliferation was assessed using a methylthiazolyldiphenyl-tetrazolium bromide (ST316, Beyotime, China) assay. A total of 1500 A2058 or MV3 cells/well were seeded in 96-well plates. Ten microliters of MTT (5 mg/ml) reagent was added to each well and incubated for 4 hours. Then, the medium was replaced with 150 μl of DMSO. The absorbance was detected at 570 nm by TECAN (Mechelen, Belgium).

### Colony formation assay

Cells were seeded in 6-well plates (800/well) and cultured for two weeks. Then, the cells were washed, fixed and stained with 0.1% crystal violet. The colonies were captured using a digital camera and counted.

### RNA immunoprecipitation (RIP) assay

RNA immunoprecipitation assays were performed by using a RIP assay kit (RIP-12RXN, Millipore, USA) according to the manufacturer’s instructions. The assay was performed in A2058 and MV3 cells. The results of the co-IP examined by Western blotting proved that the immunoprecipitation complex formed specifically with the anti-flag antibody. The RNA present in the anti-flag/RNA complexes was detected by RT-PCR.

### Tumor xenograft model

All animal experiments were approved by the Animal Ethics Committee of Zhongshan Hospital affiliated with Fudan University. A total of 8 male BALB/c nude mice (4–6 weeks; 15–18 g) were purchased from SLAC Laboratory and were maintained in a SPF facility. To detect the effects of DDX52 on tumor growth, 5 × 106 cells were subcutaneously injected into the lower flank. Each mouse was randomly injected with shDDX52 or NC cells, and all the mice were sacrificed at 4 weeks postimplantation. Tumor volumes and tumor inset time were recorded twice every week. Tumor tissues were surgically removed and subjected to hematoxylin and eosin (HE) and IHC staining.

### Statistical analysis

All experiments were repeated at least three times. SPSS and GraphPad Prism 5 were utilized to analyze the experimental results. The results are presented as the mean ± SEM and were assessed by Student’s t-test. A p-value < 0.05 in all cases was considered to be statistically significant.

## Results

### DDX52 was overexpressed in MM and predicted poor clinical outcomes

**Figure 1.**
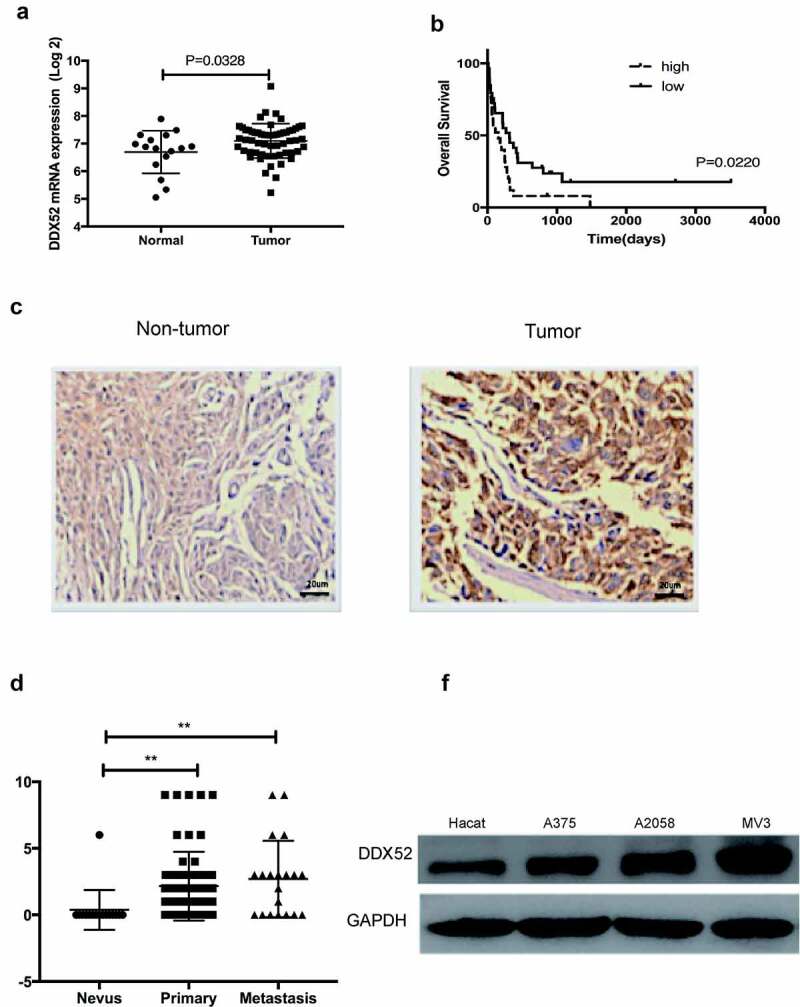
DDX52 expression was overexpressed in MM specimens and predicted poor clinical outcomes in MM patients. (a) Levels of DDX52 expression in MM tissues versus normal samples from a GEO dataset (GSE15605; Normal, n = 16; Tumor, n = 58). (b) OS curve of MM patients based on DDX52 mRNA expression from the GEO dataset (GSE22153 High, n = 25; Low, n = 29). (c) The expression levels of DDX52 in nontumor and tumor tissues were determined by immunohistochemical staining; original magnification 400 × . (d) DDX52 expression scores in tissue nevi and primary and metastatic MM tissues (nevi, n = 18; primary melanoma, n = 62; metastatic melanoma, n = 20). (e) The protein expression level of DDX52 in HaCaT and MM cells was determined by Western blot. ** P < 0.01

DDX52 has been reported to be involved in prostate and lung cancer [12,13]; however, the clinical and biological functions of DDX52 in MM are unknown. To determine the expression level of DDX52 in MM and normal tissues, we used data from the GEO database (GSE15605 and GSE22153) to assess the DDX52 mRNA level and its association with MM progression. As shown in Figure 1(a), DDX52 was overexpressed in MM compared to normal tissue (p < 0.05). Then, we further evaluated its association with patient survival in MM. The results showed that overexpression of DDX52 was associated with shorter overall survival (Figure 1(b), p = 0.022). Indeed, IHC analysis showed that DDX52 was markedly overexpressed in MM compared to nontumor tissues (Figure 1(c)). Additionally, the DDX52 level was significantly increased in primary and metastatic MM (Figure 1(d)). The protein expression of DDX52 in MM cells was significantly higher than that in nontumor cells (Figure 1(e)). Collectively, these results indicate that DDX52 is a prognostic marker for MM.

### Inhibition of DDX52 impairs cell proliferation in vitro

**Figure 2.**
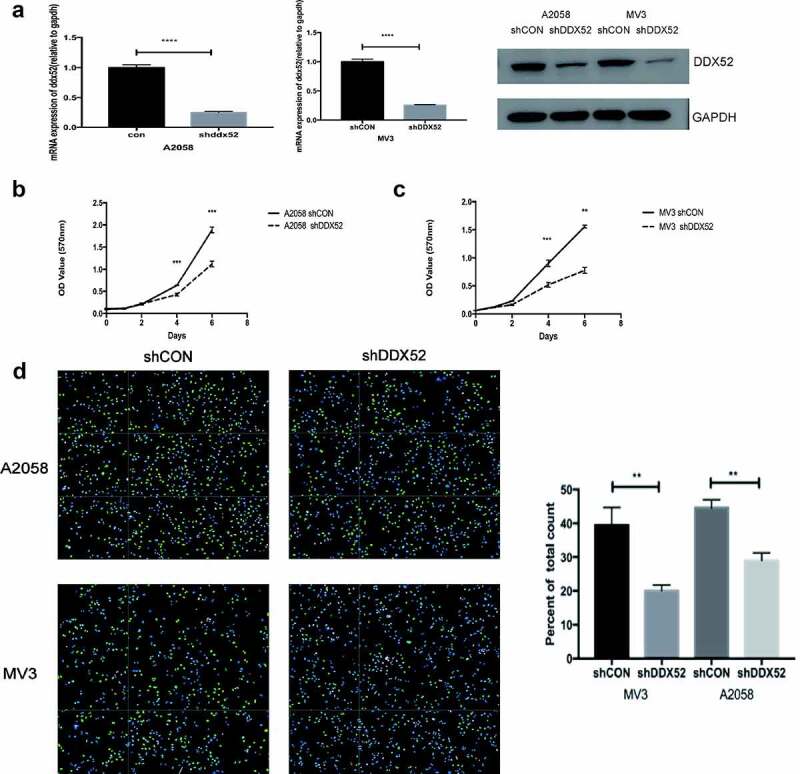
Inhibition of DDX52 impairs cell proliferation in vitro . (a) DDX52 expression in A2058 and MV3 cells transfected with shDDX52 or shCON was detected by RT-PCR and Western blotting. (b&c) The proliferation ability of shDDX52 and shCON cells was assessed by the MTT assay (OD value 570 nm). (d) EdU immunofluorescence staining of shDDX52 or shCON cells. Histograms show the percentage of EdU-positive cells. ** P < 0.01, *** P < 0.001, **** P < 0.0001

The overexpression of DDX52 in MM tissues suggests that it promotes the progression of the disease. Therefore, we constructed lentivirus-mediated stable expression of sh-DDX52 in A2058 and MV3 cells. The knockdown rates were > 70% as determined by RT-PCR and Western blotting (Figure 2(a)). The effect of DDX52 downregulation was assessed by performing an MTT assay. As shown in Figure 2(b,c), the proliferation rate of sh-DDX52-transfected A2058 and MV3 cells was markedly slower than that of the respective control cells. Additionally, suppression of DDX52 markedly reduced the number of EdU-positive cells in sh-DDX52-transfected A2058 and MV3 cells compared to the respective control groups (Figure 2(d)). Therefore, inhibition of DDX52 suppressed MM cell proliferation.

### Knockdown of DDX52 inhibited cell colony formation and tumor xenografts

**Figure 3.**
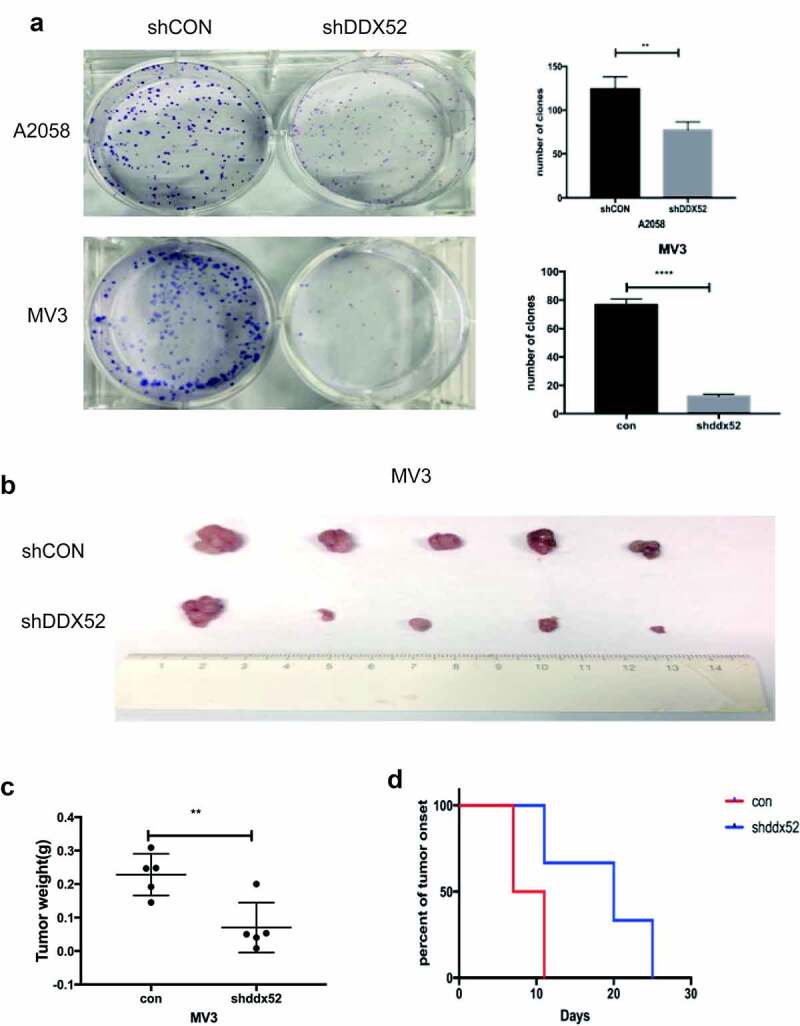
Inhibition of DDX52 impairs cell colony formation and xenograft tumor growth. (a) The colony formation ability of A2058 and MV3 cells with DDX52 knockdown was detected.. (b) Images of tumors derived from nude mice implanted with shDDX52 or shCON MV3 cells. (c) Average tumor weight of the shDDX52 and shCON groups. (d) Kaplan-Meier analysis of tumor onset. ** P < 0.01, **** P < 0.0001

As shown in Figure 3(a), the rate of colony formation was decreased in sh-DDX52-transfected A2058 and MV3 cells compared to the control cells. We used a tumor xenograft model to examine the mechanism by which downregulation of DDX52 inhibits MM cell proliferation. Knockdown of DDX52 significantly suppressed tumor growth and size (Figure 3(b)). The average tumor weight of the knockdown group was more than twofold lower than that of the control group (Figure 3(c)). Moreover, DDX52 deficiency delayed tumor onset (Figure 3(d)). Therefore, inhibition of DDX52 suppressed MM proliferation in vivo.

### DDX52 induced c-Myc expression to promote tumor progression

**Figure 4.**
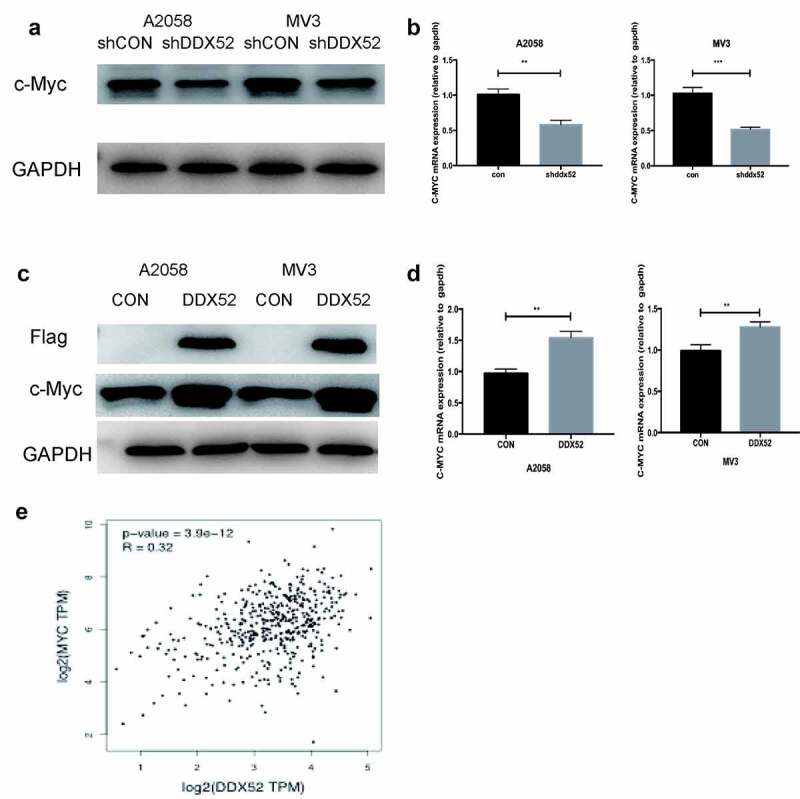
DDX52 significantly affects c-Myc expression. (a)The protein level of c-Myc was detected in shDDX52 and shCON cells. (b)The mRNA level of c-Myc was detected in shDDX52 and shCON cells. (c)The protein level of c-Myc was detected in DDX52 and CON cells. (d)The mRNA level of c-Myc was detected in DDX52 and CON cells. (e)Correlation between DDX52 and c-Myc was performed by Pearson correlation analysis using the TCGA SKCM dataset

Previous studies have shown that c-Myc is implicated in the regulation of cancer cell proliferation [16]. To explore the mechanism by which DDX52 modulates MM cell proliferation, we evaluated the expression of c-Myc in sh-DDX52-transfected A2058 and MV3 cells. The c-Myc mRNA and protein levels were decreased upon suppression of DDX52 expression (Figure 4(a,b)). Then, we evaluated the expression of c-Myc in DDX52-flag-transfected cells (Figure 4(c)). Additionally, DDX52 overexpression significantly increased the protein and mRNA levels of c-Myc (Figure 4(d)). These results were similar to those of the TCGA Pearson analysis (Figure 4(e)). These data suggest that DDX52 regulates the expression of c-Myc.

### DDX52 promotes cell proliferation by directly regulating c-Myc

**Figure 5.**
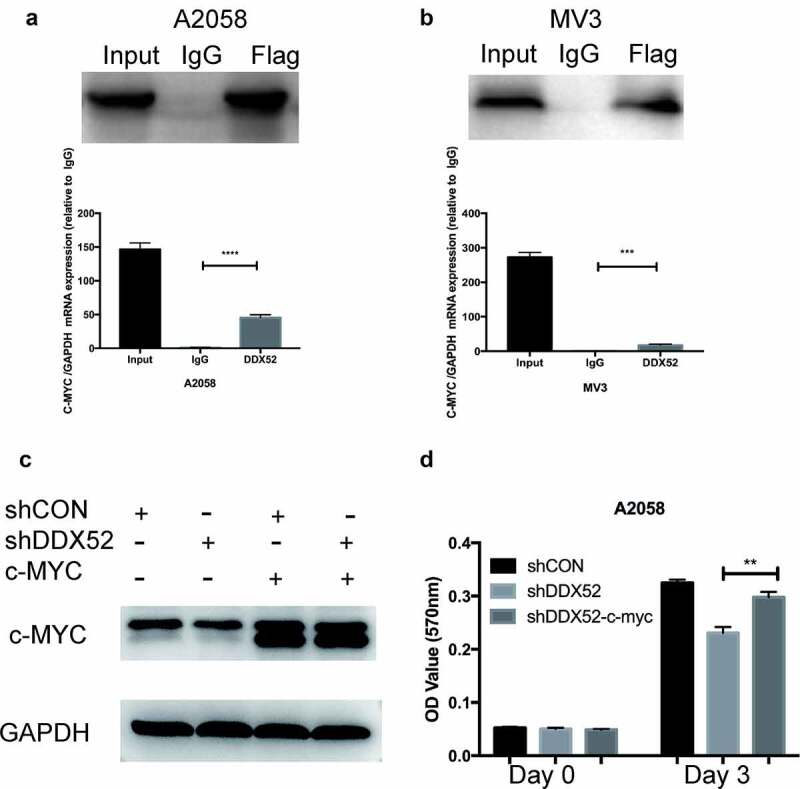
DDX52 promotes cell proliferation by directly regulating c-Myc. (a,b) RNA immunoprecipitation (RIP) assays were conducted, and that immunoprecipitates pulled down with anti-flag antibody in A2058 and MV3 cells were evaluated by Western blotting for DDX52. Beads with normal mouse IgG were used as a negative control. The levels of c-myc in DDX52-RNA complexes were detected through RT-PCR. GAPDH was utilized as a control. (c) Overexpression of c-myc in A2058 shCON and shDDX52 cells was detected by Western blotting. (d) The proliferation ability of shDDX52, shCON and shDDX52-c-Myc cells was assessed by the MTT assay (OD value 570 nm). ** P < 0.01, *** P < 0.001, **** P < 0.0001

c-Myc may be transcriptionally regulated by several RNA helicases; for example, DDX5 and DDX6 regulate c-Myc translation [17,18]. To test this hypothesis, we performed an RIP assay using DDX52-flag-overexpressing cells. The c-Myc mRNA level was more than 30-fold higher in the DDX52-flag IP group than in the IgG IP group (Figure 5(a,b)). We next restored c-Myc expression in DDX52-downregulated cells (Figure 5(c)) and examined their proliferation. As shown in Figure 5d, restoration of c-Myc expression partly rescued the proliferation deficits caused by DDX52 knockdown.

Together, these results suggest that downregulation of DDX52 suppresses tumor growth and tumorigenicity by regulating c-Myc expression.

### Discussion

Several members of the RNA helicase family function as tumor promoters in MM. For example, overexpression of DDX43 increased the RAS protein level and induced resistance to BRAF and MEK inhibition [19]. Additionally, aberrant eIF4F complex formation was increased in BRAF-resistant tumors, and inhibition of this complex synergized with a BRAF inhibitor and showed therapeutic promise for drug-resistant tumors [20]. Additionally, eIF4A regulates STAT1 expression, and pharmacologically inhibiting eIF4A exerts an antitumor effect by decreasing PD-L1 expression [21]. Moreover, DDX3X impacts the metastatic potential and response to therapy by promoting MITF mRNA translation [22]. In this study, we report that DDX52 is overexpressed in MM tissues and that overexpression of DDX52 is associated with shorter overall survival in patients. These clinical findings indicate that DDX52 might be helpful to detect MM in the early stages and improve the prognosis and diagnosis of MM patients. Moreover, downregulation of DDX52 expression suppresses cell proliferation in vitro and causes significant regression of established tumors in vivo. All these findings indicated that DDX52 might function as a prognostic biomarker and therapeutic target for MM.

RNA helicase members usually interact with various signaling molecules and regulate many biological processes. Therefore, we aimed to identify potential molecules regulated by DDX52. The RIP assay showed that DDX52 targets c-Myc and promotes its translation, indicating that DDX52 interacts with c-Myc in MM cells. The interaction between DDX52 and c-Myc was responsible for regulating the proliferation of MM cells, which was confirmed with the MTT assay in c-Myc-overexpressing MM cells. These findings showed that DDX52 transcriptionally regulates this crucial molecule in MM.

Accumulating evidence has revealed that c-Myc contributes to the progression of cancer and is a useful biomarker that is correlated with the tumor grade and disease recurrence rate [23–25]. c-Myc plays an important role in a variety of cellular processes, including proliferation, migration, differentiation, and apoptosis [26–28]. In M14 melanoma cells, downregulation of c-Myc expression induced cycle arrest and apoptosis by increasing P27 levels [29]. In uveal melanoma, c-Myc expression is associated with resistance to interferon, paclitaxel, and doxorubicin [30,31]. Moreover, downregulation of Myc expression sensitizes melanoma cells to gamma radiation in a p53-independent manner [32]. Mahapatra developed the small-molecule drug BTYNB, which destabilizes c-Myc mRNA and inhibits the proliferation of refractory melanoma cells [33]. However, c-myc is a naturally disordered protein and lacks drug recognition sites. Therefore, finding small-molecule inhibitors that can directly target the c-myc protein has been a major problem. At present, c-myc inhibitors in the research and development stages have generally low activity. Therefore, identification of the regulatory mechanism of c-Myc expression is important for improving melanoma treatment.

RNA helicases reportedly regulate the expression of c-Myc. Taniguchi observed that DDX6 promotes the transcription of c-Myc in gastric cancer cells [17]. Wu *et al*. showed that DDX5 disrupts the G-quadruplex and is involved in transcriptional activation of Myc [18]. We found that DDX52 promotes c-Myc transcription, so inhibiting DDX52 has therapeutic potential for preventing MM progression. However, further studies are required to fully elucidate the molecular mechanisms and the specific binding sites between DDX52 and c-Myc. Therefore, elucidating the function of DDX52 could pave the way for improved MM treatment.

In summary, DDX52 is overexpressed in MM tissues and promotes progression of the disease. Therefore, elucidating the function of DDX52 could pave the way for improved MM treatment.

Conclusion

The present study provides new insights into DDX52 function in human melanoma tissues. Our data showed that downregulation of DDX52 expression suppresses cell proliferation and causes significant regression of established tumors in nude mice by targeting c-Myc, suggesting that DDX52 is as a novel therapeutic target for the management of MM.
